# Preparation and Modification of Biomass-Based Functional Rubbers for Removing Mercury(II) from Aqueous Solution

**DOI:** 10.3390/ma13030632

**Published:** 2020-01-31

**Authors:** Yurong Chen, Akram Yasin, Yagang Zhang, Xingjie Zan, Yanxia Liu, Letao Zhang

**Affiliations:** 1 Xinjiang Technical Institute of Physics and Chemistry, Chinese Academy of Sciences, Urumqi 830011, China; chenyurong@ms.xjb.ac.cn (Y.C.); akram@ms.xjb.ac.cn (A.Y.); liuyanxia@ms.xjb.ac.cn (Y.L.); zhanglt@ms.xjb.ac.cn (L.Z.); 2 University of Chinese Academy of Sciences, Beijing 100049, China; 3 Department of Chemical and Environmental Engineering, Xinjiang Institute of Engineering, Urumqi 830026, China; 4 School of Materials and Energy, University of Electronic Science and Technology of China, Chengdu 611731, China

**Keywords:** elemental sulfur, cottonseed oil, post-modification, mercury adsorption

## Abstract

Biomass-based functional rubber adsorbents were designed and prepared via inverse vulcanization and post-modification. The plant rubber was synthesized with sulfur and renewable cottonseed oil as well as various micromolecular modifiers with nitrogen-containing functional groups. Results showed that types of nitrogen-containing functional groups and dosages of modifiers had a significant impact on the adsorption capacities of the resulting polymers for Hg^2+^. Notably, when the mass ratio of 2-aminoethyl methacrylate (AEMA) to sulfur was 0.05, the resulting polymer polysulfide-co-cottonseed oil modified by AEMA (SCOA2) showed the highest adsorption capacity (343.3 mg g^−1^) among all the prepared samples. Furthermore, the Hg^2+^ removal efficiency of SCOA2 remained over 80% of its original value after five adsorption-desorption cycles. It demonstrated a promising case for utilizing cheap industrial by-products (sulfur) and renewable materials (cottonseed oil). The prepared functional rubber provides alternative approach for mercury removal in waste utilization and sustainable chemistry.

## 1. Introduction

Sustainable and green chemistry, aiming at making the best of resources, reducing the generation of waste and avoiding using harmful chemicals has gained considerable attention. Great progress has been achieved in waste utilization and environmentally-friendly synthesis routes [1,2,3,4,5,6]. Elemental sulfur is largely stockpiled because its production outstrips demand [7]. Therefore, the exploration of utilizing waste elemental sulfur from petroleum processing is very important in practical industrial production. 1,3-diisopropenyl benzene (DIB) was used to stabilize polysulfide chains in inverse vulcanization as a cross-linker [8]. Sulfur-containing polymers obtained by using various cross-linkers were reported, and these polymers showed great application potentials for solid electrodes, optical materials and heavy metal remediation [9,10,11,12,13,14,15,16]. In order to meet the needs of green and sustainable chemistry, it would be desirable to employ renewable and sustainable bio-based materials to combine with sulfur for preparing functional materials. Bio-based resources are ideal due to their inexpensiveness, availability in large quantities and sustainability features [17,18]. Vegetable oils, one of the most abundant natural biomasses, contain rich reactive groups such as carbon–carbon double bonds which can react with sulfur free radical of polysulfide chain ends via radical addition [19]. These carbon–carbon double bonds are very useful functional motifs as cross-linkers for inverse vulcanization process to synthesise various high-sulfur-content functional polymers. Along these lines, in the work reported here, we choose low-cost waste sulfur from petroleum processing as functional feedstock and bio-based renewable oil resource—cottonseed oil as an organic cross-linker to prepare sulfur-containing functional polymers.

Mercury has seriously impacted on environment and human health due to its high toxicity, bio-accumulation and non-biodegradability [20]. Delegates from more than 140 countries reached an agreement on the Minamata Convention on Mercury pollution control in 2013. The treatment remediation of mercury pollution has become a global and urgent issue [21]. In the past few years, functional materials for removing mercury ions have been reported. The prepared materials include inorganic magnetic nanoparticles (such as graphene oxide-carbon composite (GO-CC), Ag/Graphene composite) [22,23], chitosan derivatives (such as formaldehyde cross-linked chitosan-thioglyceraldehyde Schiff’s base (CSTG), chitosan-phenylthiourea (Chit-PTU), glutaraldehyde cross-linked magnetic chitosan (CSm), thiourea-co-ethylenediamine modified chitosan (TC-EDA-CS) and hyperbranched polyethylenimine functionalized carboxymethyl chitosan composite (HPFC)) [24,25,26,27,28], sulfur-containing polymers or composites (such as polysulfide-co-vegetable oils, sulfur-limonene polysulfide, ZnS nanocrystal composite, Fe_3_O_4_ @SiO_2_-SH composite) [17,29,30,31], polyamine compounds (such as porous cellulose carrier modified by polyethyleneimine (Cell-PEI), polyaniline, polythioamides) [32,33,34]. Among these adsorbents, inorganic magnetic nanoparticles and sulfur-containing composites showed the lower adsorption capacities while chitosan derivatives and polyamine compounds demonstrated relatively higher adsorption capacities. However, chitosan derivatives and polyamine compounds are susceptible to the adsorption conditions including ions strength and pH values. By contrast, in view of the availability and sustainability of vegetable oil, the adequate utilization for industrial waste sulfur and the stability of polysulfide-co-vegetable oils, sulfur-containing polymers based on low-cost renewable vegetable oils are more viable. However, the sulfur-containing polymers themselves had the lower adsorption capacities for mercury ions [17,29].

It had been found that polymers with ligands such as nitrogen-containing functional groups demonstrated significantly improved abilities in mercury binding [28,32,33,34]. Besides, it was reported that when organic cross-linkers combined with elemental sulfur to form polymers through inverse vulcanization, less energy was required to cleave S-S bonds in linearly arranged polysulfide chains, thus resulted sulfur radicals can further initiate the polymerization of vinyl or allyl for post-modifying polysulfide [35,36]. The strategy of post-modification provides the possibility for introducing specific functional groups into plant rubbers formed by elemental sulfur and vegetable oils. 

In order to make the best of waste elemental sulfur and renewable cottonseed oil to prepare biomass-based functional rubber as effective adsorbents for mercury adsorption, polysulfide-co-cottonseed oil with significantly improved adsorption capacity for mercury ions through post-modification was designed. Specifically, cottonseed oil was chosen as an organic cross-linker to couple sulfur through solvent-free and atom economic inverse vulcanization process. Furthermore, micromolecular modifiers containing double bonds and nitrogen-containing functional groups were introduced into the plant rubber polymer via post-modification in order to enhance adsorption capacities of materials for mercury ions.

## 2. Materials and Methods 

### 2.1. Materials

Cottonseed oil (CO, food grade, Shihezi Kanglong oil Industry and Trade Company, Shihezi, Xinjiang, China), sulfur (powder, ≥99.5%, Tianjin Baishi Chemical Industry Co. Ltd., Tianjin, China), 2-vinylpyridine (2-VP, ≥96%, Adamas Reagent Co. Ltd., Shanghai, China), 4-vinyl pyridine (4-VP, ≥99%, Adamas Reagent Co. Ltd., Shanghai, China), 2-amino ethyl methacrylate hydrochloride (AEMA, ≥90%, Acros Organics, Shanghai, China), ethyl(E)-3-(methyl amino)but-2-enoate (EMAB, ≥95% Shanghai Accela Chem Bio Co. Ltd., Shanghai, China) and 2-(dimethylamino)ethyl methacrylate (DMAEMA, ≥99%, Adamas Reagent Co. Ltd., Shanghai, China) were used as received for chemical synthesis. Mercury(II) chloride (HgCl_2_, ≥99%, Tianjin Baishi Chemical Industry Co. Ltd., Tianjin, China), sodium hydroxide (NaOH, ≥99.7%, Tianjin Baishi Chemical Industry Co. Ltd., Tianjin, China), hydrochloric acid (HCl, ≤36%, Shanghai Hongyan Chemical Industry Co. Ltd., Shanghai, China) and nitric acid (HNO_3_, ≤65%, Xilong Chemical Industry Co. Ltd., Zhengzhou, Henan, China) were used as received for adsorption experiment.

### 2.2. Preparation of Polysulfide-Co-Cottonseed Oil (SCO) 

About 10.0 g of elemental sulfur powder and 10.0 g of cottonseed oil were charged into a 50 mL vial equipped with a magnetic stir bar. The mixture was stirred thoroughly at 150 °C in order that the feed stocks was mixed evenly. After half an hour, the mixture got gelation and formed brown rubber-like bulk polysulfide-co-cottonseed oil (SCO). Besides, the brown rubber-like SCO was crushed into particles (<1 mm) at room temperature before using for mercury adsorption.

### 2.3. Preparation of Post-Modified SCO 

**Scheme 1:**
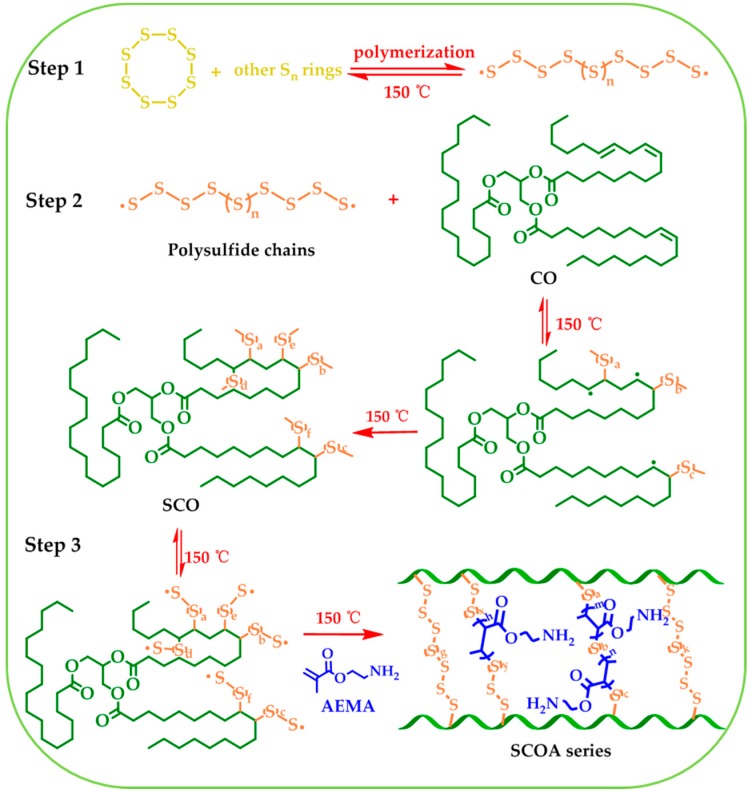
The mechanism of reaction between elemental sulfur, cottonseed oil (CO) and 2-aminoethyl methacrylate (AEMA).

When 10.0 g of elemental sulfur powder and 10.0 g of cottonseed oil were mixed under stirring at 150 °C, a certain amount of modifiers was added into the mixture, and the reactants were kept vigorously stirring for 15–40 min at 150 °C to obtain final products (specific synthesis route and conditions were shown in Scheme 1 and Table S1, respectively). The resulting dark brown rubber-like materials included SCO modified by 2-VP (SCO2V series), SCO modified by 4-VP (SCO4V series), SCO modified by AEMA (SCOA series), SCO modified by EMAB (SCOE series) and SCO modified by DMAEMA (SCODM series). The prepared materials were crushed into particles (< 1 mm) at room temperature before adsorption experiments.

### 2.4. Characterization

The morphological analysis of the prepared samples was carried out on a field emission scanning electron microscope (FE-SEM, SUPRA 55vp, ZEISS, Oberkochen, Baden-Württemberg, Germany) with an Oxford detector, operating with 2.00 kV electron beams. Fourier transform infrared spectra (FT-IR) was recorded on a spectrophotometer (VERTEX-70, Bruker, Karlsruhe, Baden-Württemberg, Germany) by using the ATR method with wave numbers ranging from 400 to 4000 cm^−1^. Elemental analysis was performed on an elemental analyzer (vario EL cube, Elementar, Langenselbold, Hessen, Germany). The specific surface area, total pore volume and pore diameter distribution were measured on a nitrogen absorption/desorption measurement (Autosorb-IQ-MP, Quantachrome, Boynton, FL, USA) and calculated by the Brunauer–Emmert–Teller (BET) equation and the Barrett–Joyner–Halenda (BJH) method. X-ray photoelectron spectroscopy (XPS) was obtained on a X-ray photoelectron spectrometer (EscaLab 250Xi, Thermo Fisher, Waltham, MA, USA) with a monochromatic Al Kα as an excitation source. 

### 2.5. Batch Mercury Adsorption Experiments

Batch adsorption experiments were carried out with three replicates to examine the removal abilities of the prepared samples for Hg^2+^ in aqueous solution at various adsorption time and Hg^2+^ initial concentration. Before adsorption experiments, crushed rubber particles were washed by using 0.1 mol L^−1^ NaOH aqueous solution to ensure total removal of residual modifiers as well as gaseous by-products derived from inverse vulcanization such as H_2_S that might interfere with adsorption experiments [17,37]. An amount of 8.0 g of adsorbent particles was added to a 150 mL sample vial containing 80 mL 0.1 mol L^−1^ NaOH aqueous solution and then the mixture was stirred thoroughly for 90 min at room temperature. Subsequently, the particles were filtrated by a 0.45 μm membrane filter and washed by ultrapure water. Then the materials were dried in air for 24 h before adsorption experiments. In a typical test, 30 mg of adsorbent particles was added to a 20 mL sample vial containing 10 mL 358.5 mg L^−1^ Hg^2+^ solution. The pH of the dilute solution was adjusted to 6.5 with 0.1 mol L^−1^ HCl or NaOH aqueous solution. The suspension solution was shaken in a thermostatic shaker (120 rpm, 30 °C) for 10 h to allow adsorption to reach equilibrium. Subsequently, the suspension solution was filtrated and the filtrate was diluted with 5% nitric acid solution into for the subsequent detection. Residual concentration of Hg^2+^ in the dilute solution was quantified by an inductively coupled plasma atomic emission spectrometer (ICP-5000, FPI, Hangzhou, China) with the limit of detection of 0.5 mg L^−1^ for Hg^2+^.

### 2.6. The Calculation of Adsorption Capacities of the Related Control Samples

The adsorption capacity of adsorbent for Hg^2+^ was calculated according to the following Equation (1) [27]:

(1) $$Q_{e}=\frac{{V(c}_{0}{-c}_{e})}{m}$$

where Q_e_ (mg g^−1^) represents the equilibrium adsorption capacity of adsorbent. c_0_ (mg L^−1^) is the initial concentration of Hg^2+^ in dilute solution before adsorption procedure, while c_e_ (mg L^−1^) is the equilibrium concentration of Hg^2+^ in dilute solution after adsorption procedure. V (L) is the volume of dilute solution and m (g) is the mass of the adsorbent added.

### 2.7. Adsorption Kinetic Study

In an adsorption kinetic experiment, 30 mg of adsorbent was immersed into 10 mL 358.49 mg L^−1^ Hg^2+^ solution at pH 6.5, and then the suspension solution was shaken in a thermostatic shaker (120 rpm, 30 °C). After a desired time, these adsorbent particles in the suspension solution were filtered out and the filtrate was diluted. Then, the residual concentration of Hg^2+^ in the dilute solution was measured. The related experimental data were fitted using the pseudo-first-order (Equation (2)) and the pseudo-second-order (Equation (3)) [38]:

(2) $$Q_{t}{=Q}_{e}{(1-e}^{{-k}_{1}t})$$

(3) $$\frac{t}{Q_{t}}=\frac{1}{k_{2}Q_{e}^{2}}+\frac{t}{Q_{e}}$$

where Q_t_ (mg g^−1^) is the adsorption capacity of adsorbent at time t (h). Q_e_ (mg g^−1^) is the equilibrium adsorption capacity. k_1_ (h^−1^) and k_2_ (g mg^−1^ h^−1^) are the pseudo-first-order and pseudo-second-order adsorption rate, respectively.

### 2.8. Adsorption Isotherm Study

The initial Hg^2+^ concentrations varied from 50 to 1600 mg L^−1^ at pH 6.5. In an adsorption isotherm experiment, 30 mg of adsorbent was added into the above Hg^2+^ solution (10 mL) and then the suspension solution was shaken in a thermostatic shaker (120 rpm, 30 °C) for 10 h. Subsequently, the suspension solution was filtered and the filtrate was diluted. Then, the residual concentration of Hg^2+^ in the diluent was analyzed. The related experimental data were fitted with the Langmuir (Equation (4)), Freundlich (Equation (5)) [39] and Langmuir–Freundlich equations (Equation (6)) [28]:

(4) $$Q_{e}=\frac{Q_{m}b_{L}c_{e}}{{1+b}_{L}c_{e}}$$

(5) $$Q_{e}{=K}_{F}c_{e}^{1/n_{F}}$$

(6) $$Q_{e}=\frac{Q_{m}b_{\mathrm{LF}}c_{e}^{1/n_{\mathrm{LF}}}}{{1+b}_{\mathrm{LF}}c_{e}^{1/n_{\mathrm{LF}}}}$$

where Q_e_ (mg g^−1^) is the equilibrium adsorption capacity of adsorbent while Q_m_ (mg g^−1^) is the maximum adsorption capacity. c_e_ (mg L^−1^) is the equilibrium concentration of Hg^2+^ in dilute solution after adsorption procedure. b_L_ (L mg^−1^), K_F_ (mg g^−1^) (L mg^−1^) 1/n_F_ and b_LF_ (L mg^−1^) 1/n_LF_ are the Langmuir, Freundlich and Langmuir–Freundlich binding constants related to the energy of adsorption, respectively; n_F_ and n_LF_ are the Freundlich and Langmuir–Freundlich empirical constants related to the adsorption capacity, respectively.

### 2.9. Reusability Test of Adsorbents

In one adsorption-desorption cycle, 30 mg of adsorbent was added to a 20 mL sample vial containing 10 mL 358.5 mg L^−1^ Hg^2+^ solution at pH 6.5. The suspension solution was shaken in a thermostatic shaker (120 rpm, 30 °C) for 10 h, and then the resulting suspension solution was filtrated and the filtrate was diluted. Then, the residual concentration of Hg^2+^ in the diluent was measured. The Hg^2+^ loaded adsorbents were collected, and then washed by ultrapure water and finally immersed into 10 mL 2.0 mol L^−1^ HNO_3_ solution for 12 h under stirring at 30 °C in order to regenerate adsorbents for the next adsorption cycle. The adsorption-desorption test was repeated 5 times and every test was carried out with three replicates. The reusability of adsorbents was measured by removal efficiency of adsorbents for Hg^2+^ and the removal efficiency was calculated according to the following Equation (7):

(7) $$\mathrm{RE}=\frac{\left(c_{0}{-c}_{e}\right)}{c_{0}}\times 100\%$$

## 3. Results and Discussion

### 3.1. Synthesis and Characterization

**Figure 1:**
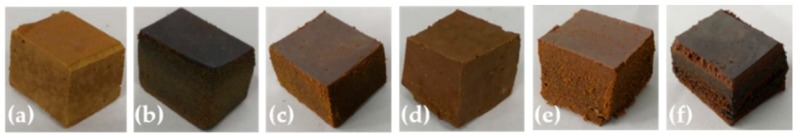
Digital photos of real sample bulk before mercury adsorption: (**a**) SCO, (**b**) SCO2V, (**c**) SCO4V, (**d**) SCOA, (**e**) SCOE, (**f**) SCODM (when mass of modifiers was 5% of sulfur mass, correspond- ing samples were chosen for taking digital photos).

**Figure 2:**
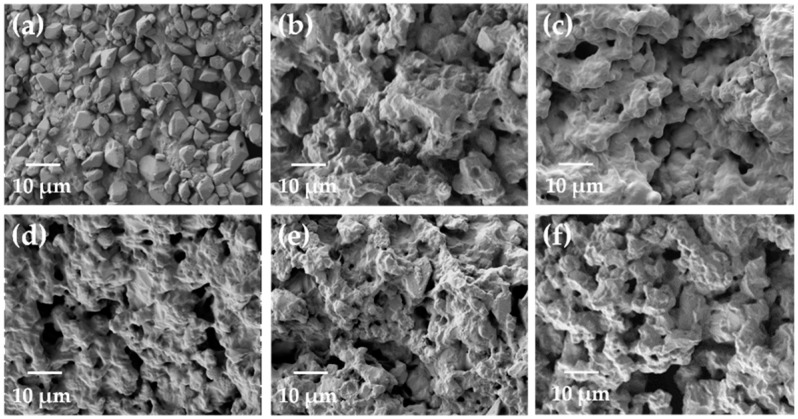
SEM images of real sample particles before mercury adsorption: (**a**) SCO, (**b**) SCO2V, (**c**) SCO4V, (**d**) SCOA, (**e**) SCOE, (**f**) SCODM (when mass of modifiers was 5% of sulfur mass, correspond-ing samples were chosen for taking SEM images).

**Figure 3:**
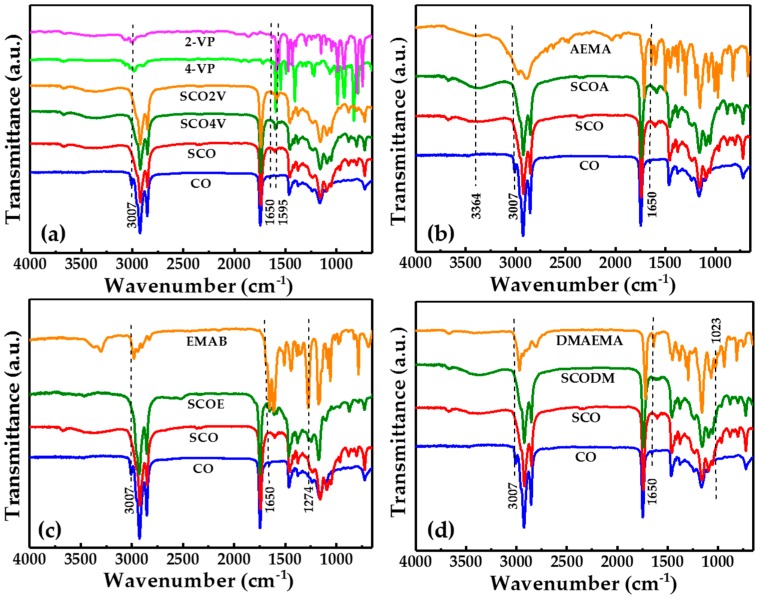
FT-IR spectrogram of the related samples before mercury adsorption: (**a**) SCO, SCO2V, SCO4V and the related cross-linker CO and modifiers 2-VP as well as 4-VP, (**b**) SCO, SCOA and the related cross-linker CO and modifier AEMA, (**c**) SCO, SCOE and the related cross-linker CO and modifier EMAB, (**d**) SCO, SCODM and the related cross-linker CO and modifier DMAEMA (when mass of modifiers was 5% of sulfur mass, corresponding samples were chosen for FT-IR analysis).

The biomass-based functional rubber adsorbents were prepared with inverse vulcanization and post-modification. The plant rubber was synthesized using waste elemental sulfur as a functional stock, cottonseed oil as an organic cross-linker and various small molecules with nitrogen-containing functional group as modifiers. The digital photos (Figure 1a–f) and SEM images (Figure 2a–f) revealed the macroscopic and microscopic surface morphology of the prepared samples. Compared with SCO, post-modified SCO samples appeared darker in color and rougher in surface feature. Moreover, as depicted in the step 1 and 2 of Scheme 1, the synthesis of SCO was achieved by free-radical addition reaction between elemental sulfur and cottonseed oil, and this process was similar to the post-modification reaction. Under vigorous stirring at 150 °C, sulfur and cottonseed oil were mixed and then generated SCO. The polysulfide chains decomposed under high temperature and the terminal sulfur radicals were exposed. These terminal sulfur radicals then react with double bonds of modifiers to get the post-modified SCO samples (as shown in the step 3 of Scheme 1). Scheme 1 illustrates the mechanism of reaction between sulfur, cottonseed oil and AEMA. Likewise, this mechanism is also applicable for reaction between sulfur, cottonseed oil and other modifiers including EMAB, DMAEMA, 2-VP as well as 4-VP were similar to this. Figure 3 shows the FT-IR spectral analysis for products. In comparison with the raw CO, 2-VP, 4-VP, AEMA, EMAB and DMAEMA, spectrogram of SCO and post-modified SCO samples all displayed that the moderate-strength peak at 3007 cm^−1^ and the weak peak at 1650 cm^−1^ disappeared. The absence of the two peaks were attributed to the vanishing of =C–H and C=C bonds stretching vibration in cottonseed oil [7] as well as modifiers during the addition reaction [19]. Figure 3a shows the strength of the peaks of SCO2V and SCO4V at around 1595 cm^−1^ enhanced notably, which were due to the participation of C=N bonds stretching vibration derived from the raw 2-VP and 4-VP [40]. Furthermore, according to Figure 3b, a new peak in SCOA was observed at around 3364 cm^−1^, which was ascribed to N–H bonds stretching vibration of AEMA. Finally, in Figure 3c,d, the two weak peaks at around 1274 cm^−1^ and 1023 cm^−1^ were proposed from C–N bonds stretching vibration of SCOE and SCODM, respectively. The changes of color, surface morphology and FT-IR spectrogram of the prepared samples implied the success of the addition reaction between sulfur and cottonseed oil as well as accomplishment of post-modification for SCO. As an additional information, gelation of products became gradually difficult with the increasing of modifiers dosage. As depicted in Table S1, the dosage of AEMA, EMAB, DMAEMA, 2-VP and 4-VP were only up to 40%, 40%, 20%, 20% and 10% (equivalent to 40%, 20% and 10% of sulfur mass), respectively. In addition, the time of gelation was also gradually prolonged with the increasing of modifiers dosage except for SCOA. These results might relate to the physical properties of modifiers.

### 3.2. Effect of Types of Nitrogen-Containing Groups in Modifiers on Adsorption Capacities of Samples

**Figure 4:**
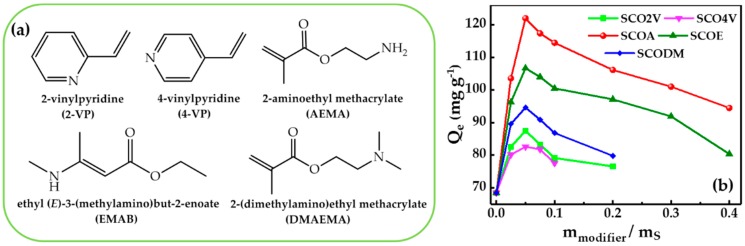
(**a**) The structures of modifiers for post-modifying SCO, (**b**) effect of types of nitrogen-containing groups on adsorption capacities of samples for Hg^2+^ (C_0_ = 358.49 mg L^−1^, sample dosage = 30 mg/10 mL, temperature = 30 °C, pH = 6.5, adsorption time = 10 h).

In order to better understand the effect of amino types of modifiers on adsorption capacities of the prepared samples, representative modifiers containing primary amino, secondary amino, tertiary amino and imine groups were chosen for post-modification of SCO. The molecular structures of different modifiers were shown in Figure 4a. As shown in Figure S1, the color of SCO and post-modified SCO samples particles became darker after mercury adsorption, which implied the effective adsorption process between adsorbents and Hg^2+^. According to Figure 4b, the adsorption capacities of post-modified SCO samples were higher than that of SCO, which indicated that the introduction of nitrogen-containing functional groups could improve adsorption capacities of these samples for Hg^2+^ to some extent. Besides, it was also observed from Figure 4b that adsorption capacities of SCOA serial samples containing primary amino group were higher than that of other post-modified SCO samples when mass ratio of corresponding modifier to sulfur was same. This implied that the primary amino group could increase the adsorption capacities of samples for Hg^2+^ more effectively. In order to answer whether content of elements was related to adsorption capacities of SCO and post-modified SCO samples containing different amino types, SCO, SCOA2, SCOE2, SCODM2, SCO2V2 and SCO4V2 were chosen for elemental analysis when the mass ratio of corresponding modifiers to sulfur was 0.05. As shown in Table 1, content of N in post-modified SCO samples was higher than that of in SCO, and this change was in line with the change of adsorption capacities of post-modified SCO samples were higher than that of SCO. Meanwhile, from SCOA2 to SCO4V2, content of N declined while content of S went up. This variation of N content was the same with the changing trend of adsorption capacities from SCOA2 to SCO4V2, whereas the variation of S content was the opposite to the changing trend, indicating that content of N and S may have important effects on adsorption capacities of samples for Hg^2+^. Moreover, the specific surface area of the related samples was tested using the BET method to investigate the correlation between specific surface area and adsorption capacity. According to the data in Table 2, specific surface areas of post-modified SCO samples were larger than that of SCO, which implied the introduction of modifiers could increase the specific surface area of post-modified samples. However, compared with the notable variation of adsorption capacities of post-modified samples, the changes of their specific surface areas were not obvious when mass ratio of modifiers to sulfur was 0.05. This implied specific surface area was not the key factor resulting in the notable variation of adsorption capacities of post-modified SCO samples.

### 3.3. Effect of Dosage of AEMA on Adsorption Capacities of SCOA Serial Samples

**Figure 5:**
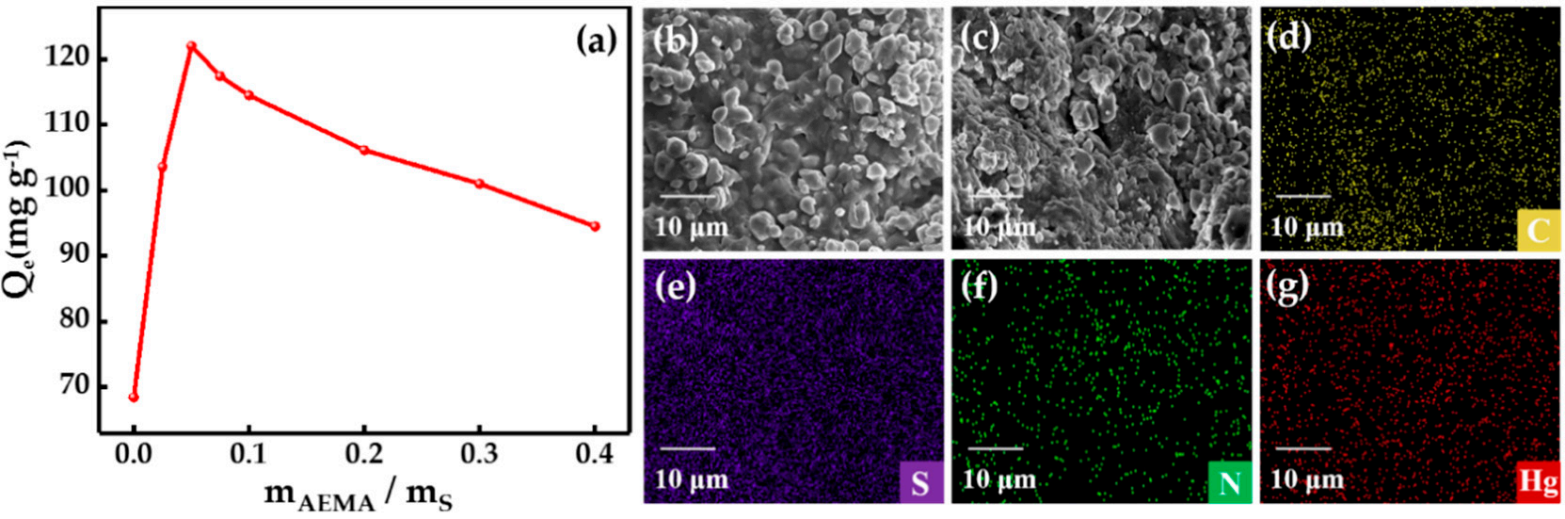
The curve of adsorption capacities of the related samples for Hg^2+^ versus mass ratio of AEMA to sulfur (**a**), SEM images of SCOA2 before mercury adsorption (**b**) and after mercury adsorption (**c**), and the corresponding EDS mapping of C (**d**), S (**e**), N (**f**), Hg (**g**) (C_0_ = 358.49 mg L^−1^, sample dosage = 30 mg/10 mL, temperature = 30 °C, pH = 6.5, adsorption time = 10 h).

Figure 4b showed that the dosage of modifiers had a significant impact on the adsorption capacities of samples. Adsorption capacities of all post-modified SCO samples increased firstly with the increasing of mass ratio of modifiers to sulfur and then decreased. When the mass ratio of modifiers to sulfur was 0.05, adsorption capacities of post-modified SCO samples reached the maximum. In order to probe the effect of modifiers dosage on adsorption capacities, representative SCOA serial samples were chosen for the next analysis. Figure 5a depicted the variation of adsorption capacities of SCOA serial samples versus mass ratio of AEMA to sulfur. When the mass ratio of AEMA to sulfur was 0.05, the adsorption capacity of SCOA2 reached the maximum of 122.0 mg g^−1^. Figure 5b shows the SEM images of SCOA2 before and after adsorbing Hg^2+^ and corresponding EDS mapping. EDS mapping revealed all elements including C, S, N, Hg elements distributed on the surface of SCOA2 homogeneously. According to Table 3, the content of N of SCOA serial samples were higher than that of SCO and increased gradually with the augment of AEMA dosage whereas the content of S declined. Interestingly, the adsorption capacities of SCOA samples did not increase with the augment of AEMA dosage all the time, but increased at first and then decreased. Moreover, according to Table 4, the introduction of AEMA increased the specific surface area of SCOA serial samples compared with SCO. However, compared with notable variation of adsorption capacities of SCOA serial samples, variation of their specific surfaces was not obvious while AEMA dosage was added little by little. The phenomenon was similar to the previous results about specific surface area analysis for post-modified SCO samples including SCOA2, SCOE2, SCODE2, SCO2V2 and SCO4V2, which maybe also imply specific surface area could have effects on adsorption capacities of post-modified samples to a certain extent but not the dominant factor causing the significant variation of adsorption capacities between them. Moreover, Figure S2a,b depicted the N_2_ adsorption-desorption isotherm and the pore diameter distribution of SCOA2, respectively. According to IUPAC classification, the isotherm of SCOA2 exhibited a typical type-IV curve (Figure S2a), which indicated the adsorption behavior of SCOA2 for Hg^2+^ involved monolayer and multilayer adsorption [28]. Figure S2b displayed that the pore diameters of SCOA2 mainly ranged from 0.5 to 3.5 nm. Although SCOA2 primarily occupied small pore sizes, the adsorbed volume was also low, which implied that pore amount in SCOA2 was limited.

### 3.4. Adsorption Mechanism

In order to better investigate the interaction between Hg^2+^ and the prepared samples, SCOA2 was chosen for the following a series of analysis because it had the maximal adsorption capacity in the previous experiments. 

#### 3.4.1. FT-IR Analysis

**Figure 6:**
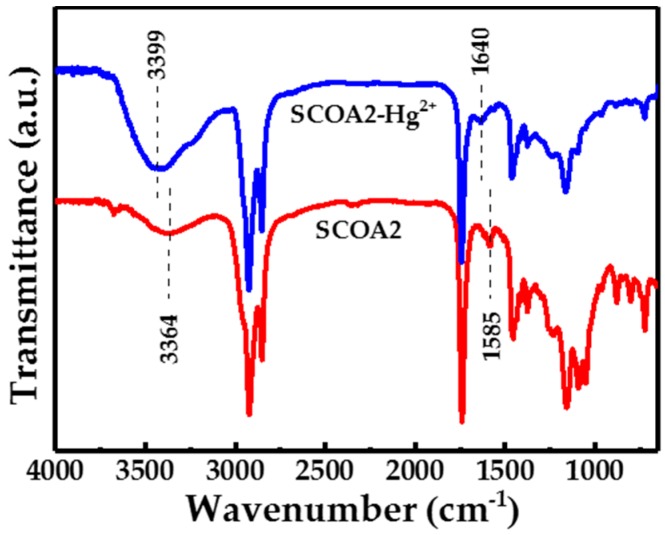
FT-IR spectrum of SCOA2 before and after mercury adsorption (C_0_ = 358.49 mg L^−1^, sample dosage = 30 mg/10 mL, temperature = 30 °C, pH = 6.5, adsorption time = 10 h).

Before and after mercury adsorption, SCOA2 was analyzed by FT-IR (Figure 6). As shown in Figure 6, after mercury adsorption, the shifting of –NH_2_ stretching vibration peak from 3364 cm^−1^ to 3399 cm^−1^ implied that the primary amine interacted with Hg^2+^ in the adsorption process. Another evidence was that the bending vibration peak of N–H shifted from 1585 cm^−1^ to higher wavenumber (1640 cm^−1^). 

#### 3.4.2. XPS Analysis

**Figure 7:**
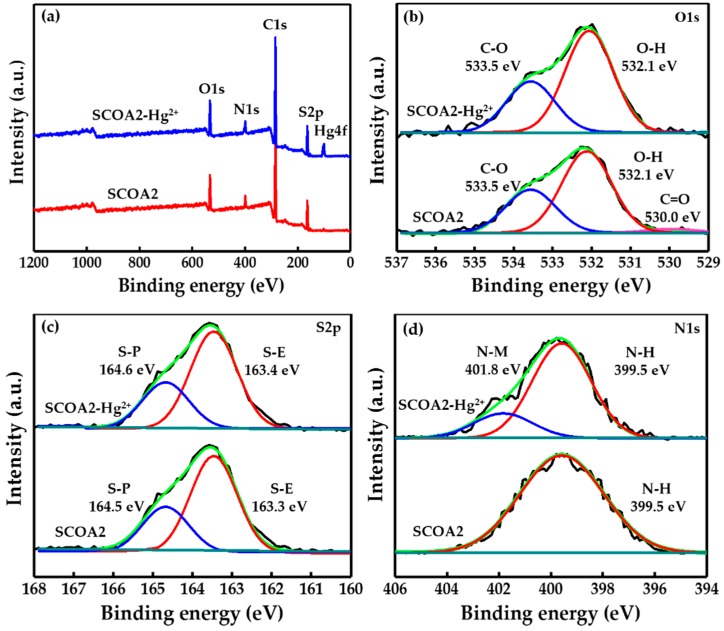
XPS spectra: (**a**) survey spectra of SCOA2 before and after mercury adsorption, high-resolution spectra of O1s (**b**), S2p (**c**) and N1s (**d**) of SCOA2 before and after mercury adsorption (C_0_ = 358.49 mg L^−1^, sample dosage = 30 mg/10 mL, temperature = 30 °C, pH = 6.5, adsorption time = 10 h).

Moreover, before and after mercury adsorption, SCOA2 was analyzed by XPS (Figure 7). As depicted in Figure 7a, the XPS survey spectra revealed the presence of carbon, nitrogen, oxygen and sulfur at ~285, ~399, ~533 eV and ~164 eV, corresponding to the C1s, N1s, O1s and S2p orbital, respectively [41,42]. In addition, after mercury adsorption, a strong peak of Hg at ~103 eV appeared in the survey spectra. The survey spectra revealed that the introduction of AEMA was helpful for the adsorption of SCOA2 for Hg^2+^. The high-resolution spectrum of O1s in Figure 7b could be fitted by three peaks at around 533.5 eV, 532.1 eV and 530.0 eV, assigning to –C=O, –OH and –C–O– respectively. For the two peaks at 533.5 eV and 532.1 eV, there was no obvious peak shift or intensity change while for the peak at 530.0 eV, the original weak peak disappeared after mercury adsorption, indicating that oxygen-containing groups of SCOA2 were rarely involved in the adsorption process. The high-resolution spectrum of S2p in Figure 7c could be fitted by two peaks at around 163.3 eV and 164.5 eV due to disulfide bonds from residual elemental sulfur (S–E) [43] and disulfide bonds from polysulfide chains (S–P), respectively. Compared with the high-resolution spectrum of O1s, the two fitting peaks also showed a minor shift after mercury adsorption. The peak at 163.3 eV shifted to 163.4 eV while the peak at 164.5 eV moved to 164.6 eV. This result implied that polysulfide chains and residual elemental sulfur in SCOA2 were possibly involved the adsorption process, which was consistent with reported work in the literature [17]. Figure 7d show the high-resolution spectrum of N1s, and the peaks at 399.5 eV before mercury adsorption could be assigned to the amine groups of SCOA2 [27]. Whereas, after mercury adsorption, a new peak appeared at 401.8 eV while the peak at 399.5 eV had no obvious changes. The new peak was possibly due to interaction between N and heavy metal ions (N–M). Previous studies showed there was a chelating interaction between N and Hg^2+^, in which the lone pair electrons from N could interact with Hg^2+^ to form the coordination bond [44]. 

**Scheme 2:**
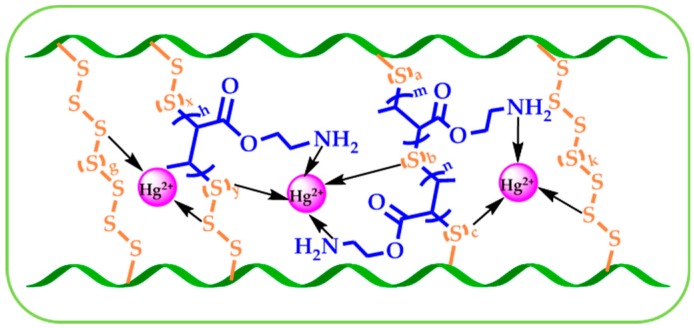
The adsorption mechanism of SCOA2 for Hg^2+^.

Based on the above FT-IR and XPS analysis, it was proposed that there were two types of interactions involved in the adsorption process of SCOA2 for Hg^2+^ (Scheme 2). One was the chelation of nitrogen-containing groups to Hg^2+^, and the other was the complexation of polysulfide chains and residual elemental sulfur to Hg^2+^. Results revealed the presence of N and S was the key that samples could adsorb Hg^2+^, which was in line with the previous results of elemental analysis for post-modified SCO samples. Whereas, the higher the content of N or S was, the larger adsorption capacities of samples were not. The content of N and S should keep an optimal ratio to ensure the best adsorption capacities of samples. This also explained the reason why adsorption capacities of SCOA serial samples did not increase along with the AEMA dosage but increased at first and then decreased.

### 3.5. Adsorption Kinetics

**Figure 8:**
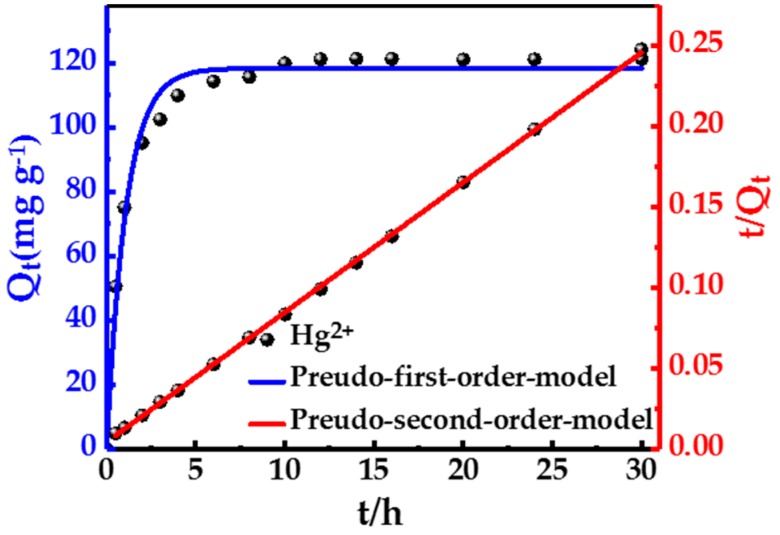
Adsorption kinetic curve of SCOA2 for Hg^2+^: pseudo-first-order-model and pseudo-second-order-model (C_0_ = 358.49 mg L^−1^, sample dosage = 30 mg/10 mL, temperature = 30 °C, pH = 6.5).

To investigate the adsorption kinetics of SCOA2, the curve of adsorption capacities of SCOA2 for Hg^2+^ versus adsorption time was plotted and the related experimental conditions and results were shown in Figure 8. In the first 2 h, for the presence of a large number of available binding sites in SCOA2, adsorption amount of SCOA2 for Hg^2+^ increased rapidly [45], and from 2 h to 10 h, the increasing slowed down because the number of available sites gradually decreased [38]. When adsorption time was prolonged from 10 h to 30 h, the adsorption amount had little change and the adsorption process reached equilibrium. Therefore, an equilibrium time of 10 h was chosen in the following adsorption experiments. To better understand the adsorption process, the pseudo-first-order and pseudo-second-order models were applied to analyze the experimental data, respectively. The related kinetic fitting parameters were listed in Table S2. As shown in Figure 8 and Table S2, the pseudo-second-order-model provided the higher regression coefficient (R^2^ = 0.9998) compared with the pseudo-first-order model (R^2^ = 0.9848). In addition, the fitting Q_t_ value (124.4 mg g^−1^) obtained from the pseudo-second-order-model also was very close to the experimental value (121.98 mg g^−1^). The results suggested the pseudo-second-order model could describe the adsorption process better, which indicated the adsorption process was controlled by chemical force [46,47]. Furthermore, the hard-soft acid-base (HSAB) theory that heavy metal ions (such as Hg^2+^) as Lewis soft acids could more easily react with Lewis soft bases (such as amine/imine) through coordination bonds also supported this conclusion [48].

### 3.6. Adsorption Isotherm

**Figure 9:**
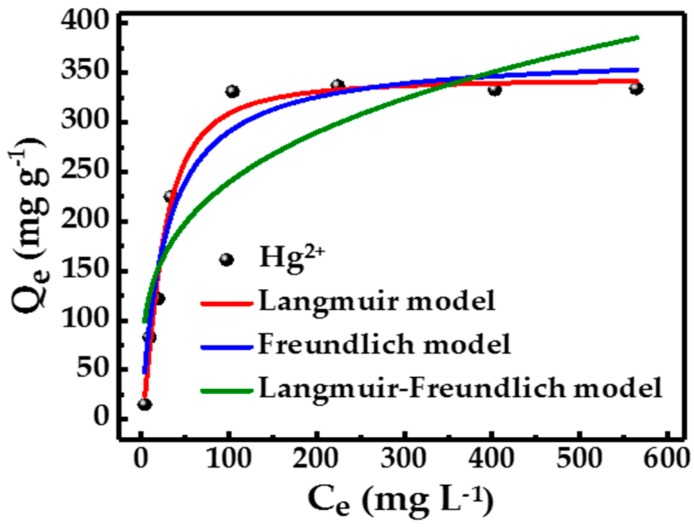
Adsorption isotherm of SCOA2 for Hg^2+^ (Sample dosage = 30 mg/10 mL, temperature = 30 °C, pH = 6.5, adsorption time = 10 h).

To evaluate adsorption ability of SCOA2 for Hg^2+^, the effect of the Hg^2+^ initial concentration on the adsorption capacities of SCOA2 was investigated, and the adsorption isotherm and the fitting curve were shown in Figure 9. With the increasing Hg^2+^ initial concentration, the adsorption capacities of SCOA2 increased gradually during the initial stage and then started to reach equilibrium at higher concentrations [39]. The fitting parameters obtained by using the Langmuir, Freundlich and Langmuir–Freundlich isotherm models were listed in Table S3. In comparison with the Langmuir and Freundlich models, the adsorption capacities of SCOA2 were better depicted by the Langmuir–Freundlich isotherm model (R^2^ = 0.9914), which implied that there was monolayer-multilayer adsorption between SCOA2 and Hg^2+^. The maximum adsorption capacity (Q_m_) of SCOA2 was 343.3 mg g^−1^. Here, some representative adsorbents which chiefly relied the chemical interaction between N or S and Hg^2+^ to play the role of adsorbents were chosen to compare with SCOA2. By contrast, SCOA2 as a biomass-based functional rubber adsorbent for removing Hg^2+^ was more attractive according to Table 5.

### 3.7. Reusability of Adsorbents

**Figure 10:**
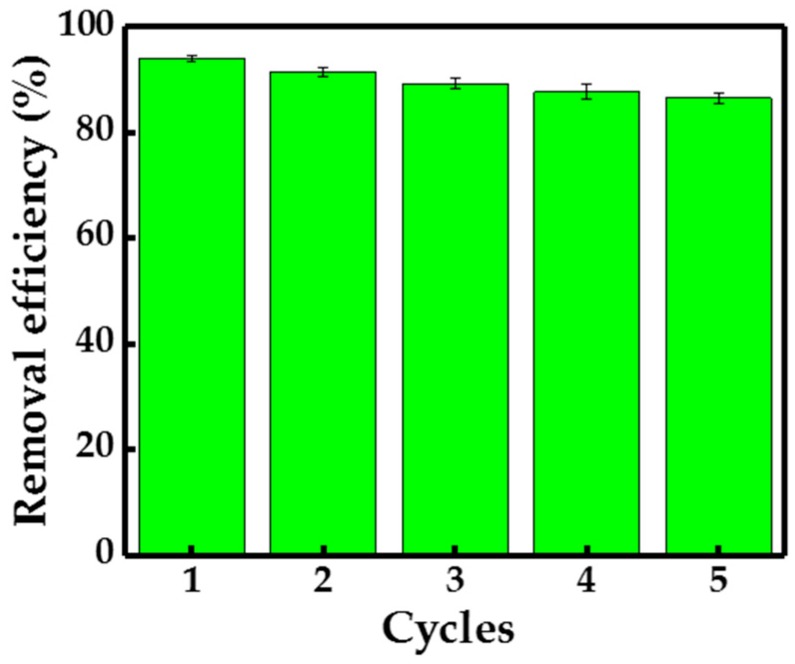
The removal efficiency of SCOA2 for Hg^2+^ during adsorption-desorption cycles (C_0_ = 358.49 mg L^−1^, sample dosage = 30 mg/10 mL, temperature = 30 °C, pH = 6.5, adsorption time = 10 h).

The adsorption-desorption experiment was practically important for evaluating the working lifetime of adsorbents. In order to investigate the reusability of SCOA2, the adsorption-desorption cycles were repeated five times. As depicted in Figure 10, after five adsorption-desorption cycles, the removal efficiency of SCOA2 for Hg^2+^ still remained over 80% of its original value. This result indicated that SCOA2 were stable, reusable and could be a potential material for mercury removal.

## 4. Conclusions

In this study, biomass-based functional rubber adsorbents for removing Hg^2+^ from aqueous solution were designed. The materials prepared successfully via solvent-free and scalable inverse vulcanization and post-modification process. The types of nitrogen-containing functional groups and dosage of modifiers showed significant impact on adsorption capacities of the resulting materials for Hg^2+^. The prepared rubber adsorbents after post-modification exhibited improved adsorption abilities for Hg^2+^—especially SCOA2, which demonstrated the best adsorption capacity (343.3 mg g^−1^) when mass ratio of AEMA to sulfur was 0.05. The adsorption process was well fitted with the pseudo-second order kinetic model and Langmuir–Freundlich isotherm model. FT-IR spectra, XPS as well as elemental analysis data revealed that the adsorption behavior was controlled dominantly by coordination between S, N and Hg^2+^. Moreover, SCOA2 showed great stability and reusability for adsorbing Hg^2+^, because its Hg^2+^ removal efficiency remained over 80% of its original value after five adsorption-desorption cycles. This work provides an attractive and promising approach for prepared value-added materials in the field of green chemistry and sustainable engineering.

## Figures and Tables

**Table 1:** The elemental compositions of SCO and post-modified SCO samples before mercury adsorption (m_modifier/_m_S_ = 0.05).

Samples	C (%)	H (%)	O (%)	N (%)	S (%)
SCO	38.88	5.721	1.983	0.006	53.41
SCOA2	38.86	5.742	2.519	0.309	52.57
SCOE2	39.06	5.728	2.081	0.241	52.89
SCODM2	38.42	5.689	2.553	0.188	53.15
SCO2V2	38.93	5.741	1.642	0.147	53.54
SCO4V2	38.88	5.692	1.424	0.114	53.89

**Table 2:** The specific surface areas, pore volumes, pore diameters and adsorption capacities of SCO and post-modified SCO samples before mercury adsorption (m_modifier_/m_S_ = 0.05).

Samples	S_BET_ (m^2^ g^−1^)	Pore Volume (cm^3^ g^−1^)	Pore Diameter (nm)	Adsorption Capacities (mg g^−1^)
SCO	591.4	0.236	0.523	68.45
SCOA2	893.6	0.196	0.524	121.98
SCOE2	892.6	0.183	0.528	106.66
SCODM2	896.1	0.188	0.519	94.58
SCO2V2	890.3	0.241	0.527	87.45
SCO4V2	894.6	0.229	0.517	82.56

**Table 3:** The elemental compositions of SCOA serial samples before mercury adsorption.

Samples	m_AEMA_/m_S_	C (%)	H (%)	O (%)	N (%)	S (%)
SCO	0	38.88	5.721	1.983	0.006	53.41
SCOA1	0.025	39.03	5.722	1.656	0.262	53.33
SCOA2	0.05	38.86	5.742	2.119	0.309	52.97
SCOA3	0.075	38.78	5.737	3.318	0.395	51.77
SCOA4	0.1	38.93	5.735	2.996	0.549	51.79
SCOA5	0.2	39.01	5.802	3.585	0.713	50.89
SCOA6	0.3	39.14	5.795	5.001	0.984	49.08
SCOA7	0.4	39.26	5.827	4.748	1.215	48.95

**Table 4:** The specific surface areas, pore volumes, pore diameters and adsorption capacities of SCOA serial samples before mercury adsorption.

Samples	S_BET_ (m^2^ g^−1^)	Pore Volume (cm^3^ g^−1^)	Pore Diameter (nm)	Adsorption Capacities (mg g^−1^)
SCO	591.356	0.236	0.523	68.45
SCOA1	883.561	0.228	0.529	103.56
SCOA2	893.605	0.196	0.524	121.98
SCOA3	884.873	0.203	0.526	117.36
SCOA4	894.586	0.189	0.518	114.45
SCOA5	889.112	0.193	0.527	106.11
SCOA6	887.563	0.209	0.521	100.99
SCOA7	895.229	0.211	0.525	94.47

**Table 5:** Adsorption capacities of different adsorbents for Hg^2+^.

Adsorbents	pH	t_e_ (min)	T (°C)	Q_m_ (mg g^−1^)	Isotherm Model	Kinetic Model	References
CSTG	5.0	60	30	98.0	Langmuir	pseudo-second-order	[24]
Fe_3_O_4_ @SiO_2_-SH	3-8	1440	25	132.0	Langmuir	pseudo-second-order	[31]
Chit-PTU	5.0	60	30	135.0	Langmuir	pseudo-second-order	[25]
CSm	5.0	200	25	152.0	Langmuir	pseudo-second-order	[26]
TC-EDA-CS	≥4.0	1440	25	217.1	Langmuir	pseudo-second-order	[27]
Cell-PEI	7.0	500	25	288.0	Langmuir	pseudo-second-order	[32]
SCOA2	6.5	600	30	343.3	Langmuir-Freundlich	pseudo-second-order	this work

## Supplementary Materials

The following are available online at https://www.mdpi.com/1996-1944/13/3/632/s1, including Table S1: synthesis conditions of biomass-based functional rubbers; Table S2: the adsorption kinetic fitting parameters of SCOA2 for Hg^2+^; Table S3: the fitting parameters of adsorption isotherm of SCOA2 for Hg^2+^; Figure S1: digital photos of real sample particles before and after mercury adsorption; Figure S2: N_2_ adsorption-desorption isotherm and pore diameter distribution curve of SCOA2.
