# Supplementary material for: Preparation and Modification of Biomass-Based Functional Rubbers for Removing Mercury(II) from Aqueous Solution
Source: Materials (Basel). 2020 Jan 31;13(3):632. doi: 10.3390/ma13030632 (PMC7040720; doi:10.3390/ma13030632)
Supplement: Supplementary file 1 [file materials-13-00632-s001.pdf]

materials

Supplementary Materials: Preparation and Modification of Biomass-based Functional Rubbers for Removing Mercury(Ⅱ) from Aqueous Solution

Yurong Chen, Akram Yasin, Yagang Zhang, Xingjie Zan, Yanxia Liu and Letao Zhang

Table S1. Synthesis conditions of biomass-based functional rubbers.

Samples	Components	Mass Ratio of Reactants	Temperature (℃)	Time of Gelation[1] (min)
SCO	S:CO	1:1	150	30
SCO2V1	S:CO:2-VP	1:1:0.025	150	20
SCO2V2	S:CO:2-VP	1:1:0.05	150	25
SCO2V3	S:CO:2-VP	1:1:0.075	150	28
SCO2V4	S:CO:2-VP	1:1:0.1	150	30
SCO2V5	S:CO:2-VP	1:1:0.2	150	38
SCO4V1	S:CO:4-VP	1:1:0.025	150	15
SCO4V2	S:CO:4-VP	1:1:0.05	150	19
SCO4V3	S:CO:4-VP	1:1:0.075	150	22
SCO4V4	S:CO:4-VP	1:1:0.1	150	28
SCOA1	S:CO:AEMA	1:1:0.025	150	30
SCOA2	S:CO:AEMA	1:1:0.05	150	25
SCOA3	S:CO:AEMA	1:1:0.075	150	20
SCOA4	S:CO:AEMA	1:1:0.1	150	18
SCOA5	S:CO:AEMA	1:1:0.2	150	18
SCOA6	S:CO:AEMA	1:1:0.3	150	15
SCOA7	S:CO:AEMA	1:1:0.4	150	15
SCOE1	S:CO:EMAB	1:1:0.025	150	20
SCOE2	S:CO:EMAB	1:1:0.05	150	25
SCOE3	S:CO:EMAB	1:1:0.075	150	28
SCOE4	S:CO:EMAB	1:1:0.1	150	30
SCOE5	S:CO:EMAB	1:1:0.2	150	32
SCOE6	S:CO:EMAB	1:1:0.3	150	35
SCOE7	S:CO:EMAB	1:1:0.4	150	38
SCODM1	S:CO:DMAEMA	1:1:0.025	150	18
SCODM2	S:CO:DMAEMA	1:1:0.05	150	25
SCODM3	S:CO:DMAEMA	1:1:0.075	150	30
SCODM4	S:CO:DMAEMA	1:1:0.1	150	36
SCODM5	S:CO:DMAEMA	1:1:0.2	150	40

Time of gelation[1] was the period from the moment that elemental sulfur and cottonseed oil were mixed fully under stirring vigorously at 150 ℃ to the moment that the mixture (including the mixture after adding modifiers) got gelation completely.

Table S2. The adsorption kinetic fitting parameters of SCOA2 for Hg^{2+}.

Model	Q_e (mg g^{-1})	k (h^{-1})	R^2
Preudo-first-order	118.4	0.9227	0.9848
Preudo-second-order	124.4	0.01454	0.9998

Table S3. The fitting parameters of adsorption isotherm of SCOA2 for Hg^{2+}.

Langmuir Model			Freundlich Model			Langmuir- Freundlich Model			
Q_m (mg g^{-1})	b_L	R^2	K_F	n_F	R^2	Q_m (mg g^{-1})	b_{LF}	n_{LF}	R^2
370.4	0.0364	0.9704	67.53	3.637	0.7956	343.3	0.0091	0.6711	0.9914

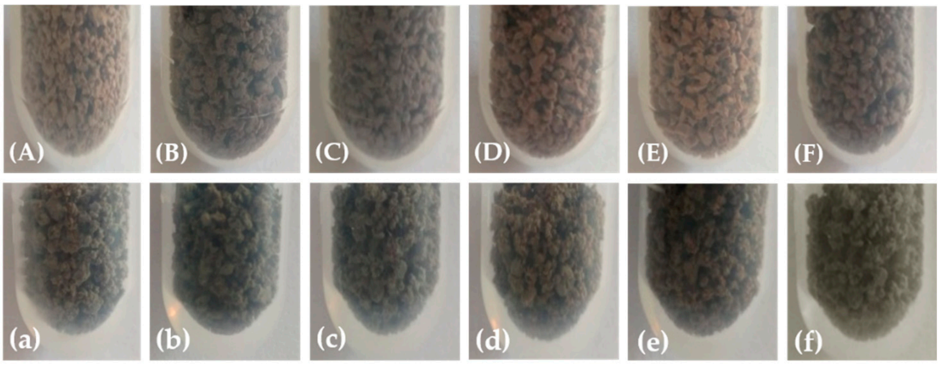

Figure S1. Digital photos of real sample particles before and after mercury adsorption: (A) SCO , (B) SCO2V, (C) SCO4V, (D) SCOA, (E) SCOE, (F) SCODM, (a) SCO-Hg^{2+} , (b) SCO2V-Hg^{2+}, (c) SCO4V-Hg^{2+}, (d) SCOA-Hg^{2+}, (e) SCOE-Hg^{2+}, (f) SCODM-Hg^{2+} (when mass of modifiers was 5% of sulfur mass, corresponding samples were chosen for taking SEM images).

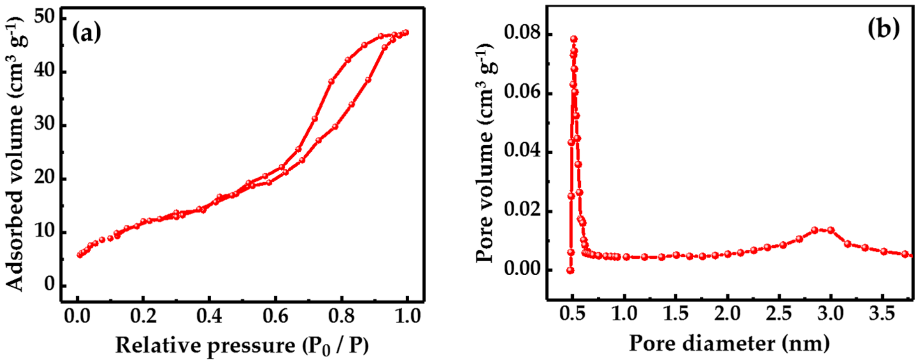

Figure S2. (**a**) N_2 adsorption-desorption isotherm of SCOA2, (**b**) pore diameter distribution curve of SCOA2.

© 2020 by the authors. Submitted for possible open access publication under the terms and conditions of the Creative Commons Attribution (CC BY) license (http://creativecommons.org/licenses/by/4.0/).
